# Retinal Capillary Nonperfusion on OCT-Angiography and Its Relationship to Kidney Function in Patients with Diabetes

**DOI:** 10.1155/2020/2473949

**Published:** 2020-11-30

**Authors:** Elysse S. Tom, Steven S. Saraf, FuPeng Wang, Qinqin Zhang, Gautam Vangipuram, Christine P. Limonte, Ian H. de Boer, Ruikang K. Wang, Kasra A. Rezaei

**Affiliations:** ^1^Department of Ophthalmology, University of Washington, Seattle, WA, USA; ^2^Department of Bioengineering, University of Washington, Seattle, WA, USA; ^3^College of Information Science and Engineering, Ocean University of China, Qingdao, China; ^4^Division of Nephrology, Department of Medicine, University of Washington, Seattle, WA, USA; ^5^Kidney Research Institute, University of Washington, Seattle, WA, USA

## Abstract

**Background:**

Diabetic retinopathy and kidney disease share underlying mechanisms of microvascular damage and are often comorbid in people with diabetes. We evaluated whether there is a relationship between retinal capillary perfusion as measured by swept-source optical coherence tomography angiography and estimated glomerular filtration rate (eGFR) and albuminuria in patients with diabetes and chronic kidney disease (CKD).

**Method:**

A cross-sectional pilot study was conducted at the University of Washington among a subset of participants with diabetes and CKD participating in a larger cohort study. Participants were excluded if they were known to have kidney disease from conditions other than diabetes. Ten participants (11 eyes) were included. Retinal nonperfusion (RNP) and vessel density (VD) were measured by swept-source optical coherence tomography angiography in 30° and 60° field of view (FOV) regions centered at the fovea. Correlations of RNP and VD with eGFR and albuminuria were analyzed.

**Results:**

Participants had a mean age of 72 years, hemoglobin A1c of 8.1%, eGFR of 45 mL/min/1.73 m^2^, and urine albumin-to-creatinine ratio of 162 mg/g. Mean (SD) RNP was 6.6% (4.2%) and 16.9% (7.7%) in 30° and 60° FOV regions, respectively. eGFR was negatively correlated to RNP in both the 30° and 60° FOV regions (*R* = −0.69, *p*=0.004, and *R* = −0.46, *p*=0.057, respectively), and correlations were stronger among a subset of 7 participants with evidence of diabetic retinopathy on exam and fundus photos. The estimated GFR was not significantly correlated with vessel density. Urine albumin-to-creatinine ratio was not significantly correlated with RNP or VD.

**Conclusions:**

Our proof-of-concept study showed that lower eGFR was significantly correlated with retinal nonperfusion in participants with diabetes and CKD. Advanced retinal imaging may enhance the noninvasive evaluation of kidney function in diabetes.

## 1. Introduction

Diabetes mellitus is a worldwide epidemic expected to affect 700 million people by the year 2045 [1]. Diabetic retinopathy and kidney disease are common microvascular complications associated with vision loss and kidney failure. Historically, epidemiologic and clinical studies have demonstrated a positive correlation between diabetic kidney disease and incidence of diabetic retinopathy [2–4], with multiple studies showing an association between higher qualitative clinical grades of diabetic retinopathy and albuminuria [5–7].

Diabetes is thought to damage the microvasculature of the retina and kidney in similar ways. While the exact molecular mechanisms of how hyperglycemia causes vascular damage are not completely understood, experimental evidence has supported a contribution from the polyol pathway, increased inflammatory oxidative stress, increased advanced glycation end products, and activation of protein kinase C in diabetic kidney disease and diabetic retinopathy [8]. In the kidney, these mechanisms lead to glomerular basement membrane thickening, podocyte detachment, and glomerulosclerosis, while in the retina they result in capillary basement membrane thickening, selective pericyte loss, and retinal nonperfusion [2, 8]. Understanding the extent of vascular injury in diabetic kidney disease may be important for subclassification and development of new treatments [9]. However, predicting histopathologic kidney changes is difficult without performing an invasive kidney biopsy, and thus examining the microvasculature noninvasively in the retina, which is similarly exposed to chronic hyperglycemia, may prove to be a useful in vivo substitute.

Chronic kidney disease (CKD) is usually diagnosed by abnormalities in functional markers while diabetic retinopathy is typically diagnosed by direct observation with ophthalmoscopy with or without fundus photographs. Until recently, there was no way to quantitatively measure the degree of ischemia or nonperfusion of the retina, so direct correlations with kidney function could be made. We now have the ability to quantitatively collect data about the retinal microvasculature using swept-source optical coherence tomography angiography (SS-OCTA), a noninvasive retinal imaging method which allows for three-dimensional examination of blood flow and vascular abnormalities at multiple layers of the retina, resolving capillary structure with a lateral resolution of 10–20 microns [10]. Prior to the establishment of OCTA, the retinal microvasculature was evaluated qualitatively with fluorescein angiography (FA), a retinal imaging modality that requires intravenous injection of dye to acquire two-dimensional images of the retinal vessels [11]. Unlike FA, OCTA does not require dye, has higher image resolution, enables quantitative analysis of the retinal vasculature with a 3-dimensional model, and is able to isolate distinct capillary layers, including the superficial capillary plexus, radial peripapillary capillary network, and deep vascular plexus [12]. OCTA is a quicker, safer, and more convenient way of imaging various layers of the retinal microvasculature.

If strong relationships exist between the degree of microvascular complications in the eyes and kidney, OCTA may prove to be a useful tool in identifying features of CKD among people with diabetes.

## 2. Materials and Methods

### 2.1. Study Design

We conducted a cross-sectional study examining the associations of retinal capillary nonperfusion (RNP) and vessel density (VD) with estimated glomerular filtration rate (eGFR) and albuminuria from 10 participants with type 2 diabetes (T2D) and CKD. Participants were recruited from the Continuous Glucose Monitoring to Assess Glycemia in Chronic Kidney Disease (CANDY) study, a prospective cohort study comparing patterns of hypoglycemia in individuals with T2D with and without CKD [13]. Participants were enrolled in the CANDY study between August 7, 2015, and July 12, 2017, and subsequently had eye imaging performed at the University of Washington Eye Institute. The study was approved by the Institutional Review Board of the University of Washington, and informed consent was obtained from each subject prior to imaging. The study followed the tenets of the Declaration of Helsinki and was in compliance with the Health Insurance Portability and Accountability Act (World Medical Association/Helsinki).

### 2.2. Study Population

The CANDY study included adults with a clinical diagnosis of T2D who were being treated with insulin or a sulfonylurea [13]. Individuals with moderate to severe CKD (eGFR 6 to <60 ml/min/1.73) were enrolled in the study. Participants who were under 18 years old, non-English speaking, kidney transplant recipients, actively receiving dialysis, pregnant, using a continuous glucose monitor, undergoing therapy for cancer, or receiving treatment with erythropoietin were excluded.

Of the patients enrolled in the CANDY study, twenty-five patients with CKD were contacted and seventeen patients presented to the clinic for imaging with SS-OCTA. Seven patients were excluded due to poor quality scans (*N* = 5) or kidney pathology other than diabetic kidney disease (*N* = 2). Specifically, one patient was excluded due to CKD related to heart transplantation and calcineurin inhibitor toxicity, and one patient was excluded due to CKD resulting from familial focal segmental glomerulosclerosis. Ten participants were included in the study. Of the 10 people included in the study, one participant had both eyes analyzed and nine participants had 1 eye analyzed due to poor quality scans of the fellow eye or limited patient tolerance to complete scanning of the fellow eye. Thus, eleven total eyes were included in the study.

### 2.3. OCT-Angiography Image Collection

SS-OCTA was performed using a 100 kHz Plex Elite 9000 device (Carl Zeiss Meditec Inc., Dublin, USA), which uses a central wavelength of 1060 nm, a bandwidth of 100 nm, a 100 kHz A-line rate, and an A-scan depth of 3 mm in tissue. The axial resolution was approximately 5 *μ*m in tissue while the lateral resolution was approximately 14 *μ*m. A scanning pattern of 12 mm x 12 mm was used to collect the wide field of view (FOV) blood flow images. In the 12 mm x 12 mm scan, 500 A-lines produced one B-scan, and B-scans were acquired at 500 different spatial locations and repeated two times at each location. This scanning protocol resulted in a uniform spacing of 24 *μ*m between pixels in the final *en face* projected OCTA images. The output datasets were processed using the complex optical microangiography (OMAG) algorithm [14–16].

### 2.4. Image Acquisition and Montage Strategy

**Figure 1:**
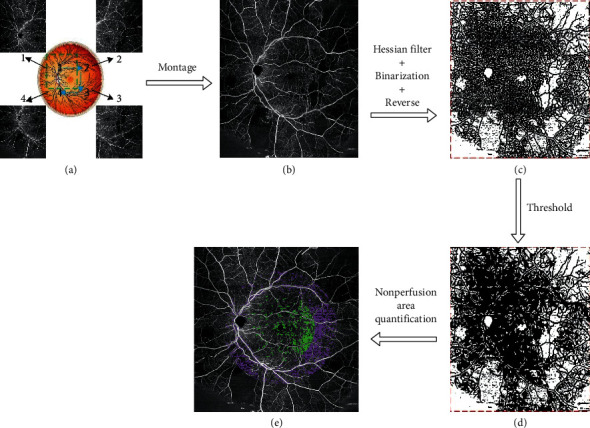
Optical coherence tomography angiography (OCTA) measurements and scanning protocol. Flowchart showing OCTA measurements and scanning protocol. (a) Four 12 mm × 12 mm scans were used to complete a wide field of view (FOV) image for each participant, (b) the original wide FOV image of retinal blood flow, (c) the nonvessel map, (d) the nonperfusion area map, and (e) the quantification results map; green: nonperfusion area in 30° FOV quantification region and purple: nonperfusion area in 60° FOV quantification region.

Base scans of 12 mm x 12 mm were obtained in this study. To achieve a wide FOV image, four base scans were performed in each retinal quadrant as labeled in Figure 1(a). A semiautomatic segmentation software was used to segment the retinal layers for further analysis [17]. The retinal layer was defined as the region from the internal limiting membrane (ILM) to the outer plexiform layer (OPL). Blood flow was observed using maximum intensity projection *en face* images. The *en face* images were montaged through an automated process to an approximately 20 mm × 20 mm image as shown in Figure 1(b).

### 2.5. Image Processing

Image processing was carried out to prepare the scan for RNP and VD analyses. A Hessian filter was applied to the montaged blood flow image to distinguish the vessel patterns. Binarization and a reversion process were performed on the output image to obtain the nonvessel map as shown in Figure 1(c). The methods of applying the Hessian filter to isolate retinal vasculature have been described elsewhere [18]. Since the image area devoid of blood vessels is not equivalent to nonperfusion area, a threshold was applied to the nonvessel map (Figure 1(c)) to remove the background and produce the true nonperfusion area map (Figure 1(d)). Areas of nonperfusion were defined as voids of more than 0.03 mm^2^ where capillaries were not detected. The value of 0.03 mm^2^ was chosen based on similar analyses of 10 normal eyes from 10 volunteers and is similar to the value found in physiologic studies investigating retinal intercapillary distance [19].

### 2.6. Calculation of Retinal Nonperfusion and Vessel Density

RNP and VD values were obtained for each eye within retinal sectors encompassing 30° and 60° centered at the fovea (Figure 1(e)). RNP was obtained by dividing the total nonperfusion area within the sector by the total area of the sector expressed as a percentage. The optic nerve head and foveal avascular zone were excluded from the analysis. VD was obtained by dividing the area occupied by vessels by the total area measured, which is expressed as a percentage.

### 2.7. Measurement of Kidney Function Biomarkers

Serum creatinine, urine albumin, and urine creatinine measurements were obtained from two study visits, which occurred approximately 3 weeks apart [13]. Estimated GFR values were calculated using the CKD-EPI equation from creatinine traceable to isotope dilution mass spectrometry [20]. The mean of the two eGFR values was calculated and used for analyses. Urine albumin and creatinine were measured using the Beckman DxC clinical chemistry analyzer. Urine ACRs were calculated, and the mean of the two values was used for analyses.

### 2.8. Clinical Covariates

Demographic (gender, age, and race) and medical history data were obtained through self-reporting (13). Hemoglobin A1c was measured from whole blood samples collected over two study visits using high-performance liquid chromatography, and the mean of these values was calculated. Diabetic retinopathy status was determined by retina specialists based on the Early Treatment Diabetic Retinopathy Study definition, which uses features seen on the exam including microaneurysms and retinal hemorrhages, venous beading, intraretinal microvascular abnormalities, and neovascular vessel growth [21]. The diagnosis was made based on clinical examination with or without fundus photography per discretion of the retina specialist.

### 2.9. Statistical Analysis

To compare the demographic data of the DR group and diabetes without retinopathy (DWR) group, the independent *t*-test was used for continuous variables, and Pearson's chi-squared test or Fisher's exact test was used for categorical variables. When both eyes were included, the generalized linear mixed model approach was adapted to adjust the correlated observations. The comparison of vessel density and nonperfusion density between the participants with DR and DWR was analyzed using the generalized linear mixed model method. Each individual eye was considered a separate data point. For correlation analyses, linear regression and linear fitting were performed. Analyses were conducted with IBM SPSS Statistics for Windows, version 26.0 (IBM Corp. Released 2019, Armonk, New York). *p* values of less than 0.05 were considered statistically significant.

## 3. Results

Eleven eyes from 10 participants were included in the study. The mean age of the ten included participants was 72 (6.9) years, the mean urine ACR was 162.1, the mean eGFR was 45.4 (SD 11.0), and the mean HgBA1c was 8.1 (SD 1.3) (Table 1). Sixty percent were male and 40% were female. Seventy percent were Caucasian, 10% were African American, and 20% reported other races; none reported Hispanic ethnicity. Six participants had DR and 4 participants did not have DR. A more detailed description of participant characteristics is summarized in Table 1.

Out of the 11 eyes, 4 eyes were left eyes and 7 eyes were right eyes. Eight eyes were pseudophakic and 3 eyes were phakic. Six eyes were characterized as mild DR, 1 eye as severe DR, and 4 eyes as DWR (40%). There were no cases of macular edema, and no eyes underwent photocoagulation. For all participants regardless of DR status, mean 30° RNP was 6.58% (SD 4.2%), mean 60° RNP was 16.9% (SD 7.71%), mean 30° VD was 45.12% (SD 1.81%), and mean 60° VD was 41.54% (SD 3.7%) (Table 2). When separated by DR status, mean 30° RNP was 4.41% (SD 1.30%) for DWR eyes and 7.81% (SD 4.91%) for DR eyes, and mean 60° RNP was 11.15% (SD 2.49%) for DWR eyes and 20.18% (SD 7.83%) for DR eyes. Furthermore, mean 30° VD was 45.55% (SD 2.52%) for DWR eyes and 44.87% (SD 1.46%) for DR eyes, and mean 60° VD was 43.28% (SD 2.48%) for DWR eyes and 40.54% (SD 3.56%) for DR eyes. These differences were not statistically significant. None of the collected participant demographics were significantly different between the two groups (DR versus DWR).

**Figure 2:**
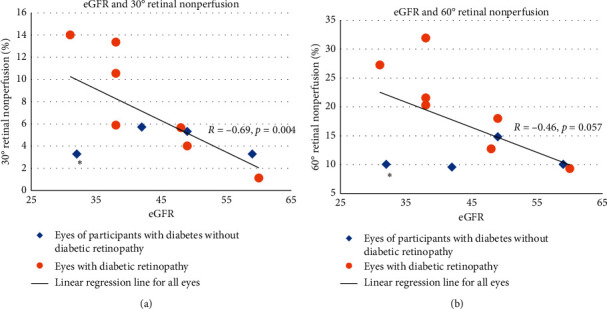
Estimated glomerular filtrate rate (eGFR) and retinal nonperfusion (RNP). Linear regression between eGFR and RNP. 30° field of view (FOV) (a) and 60° FOV (b) quantification results show a statistically significant negative correlation between eGFR and 30° retinal RNP in all participants. Diamonds represent eyes of patients with diabetes without diabetic retinopathy. Circles represent eyes with diabetic retinopathy. Solid black line shows the linear fit of all eyes. Asterisk (^*∗*^) highlights an outlier eye with low RNP and low eGFR.

As seen in Table 3 and Figure 2, when all eyes were analyzed together regardless of DR status, the negative correlation between eGFR and 30° RNP (*R* = −0.686, *p*=0.004) was statistically significant, and the association between eGFR and 60° RNP approached statistical significance (*R* = −0.457, *p*=0.057). There was no significant association between eGFR and VD. When separated by the DR status, eGFR in DR participants showed a statistically significant correlation with 30° VD (*R* = 0.81, *p*=0.027), with 30° RNP (*R* = −0.89, *p*=0.008), and with 60° RNP (*R* = −0.84, *p*=0.017). There was no significant association between urine ACR and either VD or RNP.

## 4. Discussion

In this study, we evaluated whether there is a relationship between retinal capillary perfusion as measured by OCTA and kidney function parameters including eGFR and urine ACR in people with diabetes and CKD. When all eyes meeting study criteria were analyzed collectively, a strong correlation was found between eGFR and RNP in the 30° FOV, with the same analysis in the 60° FOV approaching statistical significance. The correlation between RNP and eGFR was found to be stronger and statistically significant in both the 30° and 60° analyses when excluding eyes that did not show clinical signs of DR. Our study suggests that decreased perfusion of the retinal microvasculature in patients with T2DM is associated with CKD as measured by eGFR, and this association was stronger in eyes with DR.

Our study is a pilot for further large-scale study, which may provide better insights on the utility of OCTA to evaluate parameters of diabetic kidney disease. Nonetheless, our small-scale study found strong correlations between retinopathy and kidney disease and raised important issues for consideration in the future application of these methods. First, the correlation of RNP and eGFR was much stronger when the subset of eyes without clinical DR was excluded. There are multiple possible explanations for this finding. The number of included eyes without DR in the study was 4, which is likely too small to identify a meaningful correlation. Perhaps with larger sample size, a meaningful correlation could be found even in eyes without DR. Another possible explanation is that RNP needs to reach a certain severity threshold in order to correlate with eGFR, which may not be achieved until clinical signs of DR are apparent. Finally, diabetic kidney disease likely represents a heterogeneous group of disorders, with perhaps only some mechanisms correlating with eye disease. Since DR participants had low urine ACR, they may have had different contributing mechanisms to their kidney disease, therefore weakening the relationship. Examining eyes with DR using OCTA in conjunction with patients undergoing kidney biopsy may be one avenue of future research to investigate this possibility.

A second insight provided by our study is that OCTA parameters could potentially be used to identify patients whose kidney disease may be from multiple causes. One participant in our study who met the study criteria was a clear outlier who had no signs of clinical DR, had minimal RNP, but had the second-lowest eGFR of all included participants (see asterisks in Figure 2). Knowing that a patient's RNP is near normal with disparate eGFR may serve as a clue in clinical practice that etiologies of kidney disease should be considered other than microvascular diabetes-related injury.

The third insight provided by our study was the lack of correlation between urine ACR and either retinal capillary parameter. Since higher urine ACR is related to greater kidney damage, we expected higher associated retinal capillary loss. The reason for the absence of correlation is unclear but may be due to a small sample size. A larger-scale study may ultimately reveal a correlation or provide additional insights.

In contrast to our study, Cao et al. reported that average vessel density in the superficial capillary plexus and deep capillary plexus measured with OCT-A is not associated with creatinine in DM2 patients [22]. Perhaps the reason for the lack of association is that the study population had near-normal kidney function compared to our study's population, and creatinine is not as accurate of an estimate of kidney function as eGFR. In addition, Grunwald et al. reported that the progression of DR was associated with the progression of CKD on univariable but not multivariable analysis, indicating that similar risk factors may be affecting the progression of microvascular disease [23]. However, this study characterized the progression of DR using fundus photos that were graded by observers. OCTA is much more precise and able to quantitatively measure RNP, a feature that is not apparent on fundus photos or physical exam. As recent studies have shown, RNP can even be reduced without detectable diabetic fundus changes [24, 25]. For these reasons, we believe the implementation of OCTA in our study greatly enhances our ability to detect a correlation if one exists.

Our study reaffirms prior reports of a correlation existing between the clinical assessment of diabetic retinopathy and renal dysfunction [26, 27]. Prior studies have shown that retinal arteriolar narrowing and smaller retinal vascular fractal dimensions are found in chronic kidney disease (CKD) patients with diabetes [28, 29]. A cohort study from Taiwan previously showed that higher serum creatinine and lower eGFR were associated with the development of proliferative diabetic retinopathy detected on fundus photos [30]. In addition, a retrospective cohort study by Lee et al. found that patients with ischemic DR who demonstrated capillary nonperfusion (>10 disc areas) on FA had a higher risk of CKD progression [31]. Nagaoka et al. reported that retinal blood flow measured by laser Doppler velocimetry was negatively correlated with serum creatinine [32]. However, these studies did not examine OCTA, which has the advantage of producing quantitative data such as RNP. A study by Ting et al. has shown that renal impairment is associated with reduced capillary density index on OCTA [33] but did not investigate RNP, which in our study proved to be the parameter that demonstrated a correlation with eGFR and not VD. Furthermore, a cross-sectional study by Yang et al. found on multivariate analysis that eGFR was associated with choriocapillaris flow density on OCTA in diabetic eyes. However, the study did not compare DWR eyes to DR eyes with regard to eGFR correlation [34]. A recent study performed in China found a positive correlation between eGFR and retinal VD, but the FOV was limited to a 3 mm circle or 10° around the fovea, which is much smaller than the FOV in our study. Additionally, the range of eGFR was normal for the majority of patients, which provides limited external validity to patients with significant renal impairment [35]. Although significant vascular alterations in the central macula have been detected in people with diabetes using OCTA, more recent data suggest that these findings are more pronounced in noncentral regions and provide an additional predictive value of diabetic retinopathy status [36]. Prior traditional smaller field OCTA studies have shown that the foveal avascular zone (FAZ) in the central macula may be enlarged in diabetics, but we believe this is an inferior metric of retinal nonperfusion. Since diabetes affects the peripheral retina more severely than the central retina, the newer wide FOV OCTA can be used to evaluate peripheral retinal nonperfusion. In addition, due to the image quality of the wide FOV OCTA, FAZ measurements would be inexact and would require a reversion back to the older generation, smaller field of view OCTA images. While FA can quantify the FAZ zone, it is an invasive imaging modality, and our study used a noninvasive protocol. To our knowledge, our study is the first to assess RNP and retinal VD in wide FOV (30° and 60° FOV) OCTA images and demonstrate a correlation with eGFR in patients with diabetes. The strong correlation between retinal nonperfusion and eGFR may give us insight into the vascular component of the pathogenesis of diabetic kidney disease.

OCTA has already proven to be a useful modality in the evaluation of DR in the field of ophthalmology, and our study suggests that it may also find utility in evaluating for diabetic kidney dysfunction and assessing underlying CKD mechanisms. Due to the invasive nature and associated risks of renal biopsy, alternative modalities for assessing renal function that can predict the degree of diabetic kidney damage or detect alternative contributions to kidney dysfunction should be explored. It is possible that data obtained from OCTA could be paired with traditional kidney parameters to better understand the pathophysiology of a particular patient's renal dysfunction or better inform the decision to proceed with renal biopsy, such as in the outlier participant highlighted in Figure 2. A larger-scale study is needed to test these possibilities.

This pilot study has several limitations. The main limitation is that the study was a single-center study with small sample size. Our center's proficiency in obtaining high-quality OCTA images has improved since the time of this study. Another limitation is that our study was cross-sectional, and we were unable to look at disease progression over time. The cross-sectional design makes it impossible to determine the characteristics that account for the observed correlations, whether they are risk factors for both retina and kidney disease or confounders for the observed correlations. Lastly, we did not have any kidney tissue available to confirm whether the low eGFRs were from diabetes versus other kidney pathologies. Large-scale, prospective study including patients undergoing renal biopsy as part of their routine care would account for these limitations.

## 5. Conclusions

In conclusion, OCTA is an imaging modality with the ability to resolve retinal capillary alterations in diabetic eyes with a lateral resolution of 24 microns when using our methodology. The collected images approach the scale where they begin to resemble histology *in vivo*, which may serve as an important avenue to noninvasively collect data on the burden of systemic microvascular disease from diabetes. Our pilot study suggests that retinal vascular loss as detected on OCTA correlates with eGFR and could inform our understanding of diabetic kidney disease and its heterogeneous mechanisms. A further large-scale study is warranted to elucidate the clinical utility of these methods in the field of nephrology.

## Figures and Tables

**Table 1:** Characteristics of ten participants in the continuous glucose monitoring to assess glycemia in chronic kidney disease study with successful retinal imaging by optical coherence tomography angiography.

Demographics	
Age	72.0 (6.9)

Male	6 (60%)

*Race/ethnicity*	
White	7 (70%)
Black	1 (10%)
Others	2 (20%)

*Health history*	
Current smoking	0 (0)
History of myocardial infarction	0 (0)
History of heart failure	1 (10%)
History of stroke	1 (10%)
Duration of diabetes (years)	19.9 (6.6)

*Medication use*	
Insulin	8 (80%)
Insulin dose (units/kg/day)	0.59 (0.49)
Insulin secretagogues	4 (40%)
^*∗*^Other glucose-lowering agents	3 (30%)
Antihypertensive medications	9 (90%)
ACEi/ARBs	7 (70%)
Beta blockers	7 (70%)

Lipid-lowering medications	9 (90%)
Statins	9 (90%)

*Physical characteristics*	
Body mass index (kg/m^2^)	31.8 (4.4)
Systolic blood pressure (mm Hg)	139.3 (19.0)
Diastolic blood pressure (mm Hg)	70.9 (11.5)

*Laboratory values*	
eGFR (mL/min/1.73 m^2^)	45.4 (11.0)
Urine ACR (mg/g), median (IQR)	162.1 (68.0–626.8)
Hemoglobin A1c (%)	8.1 (1.3)

*DR status by participants*	
Yes	6 (60%)
No	4 (40%)

Entries above represent the mean (SD) for continuous variables and *n* (%) for categorical variables. ^*∗*^Other glucose-lowering agents include dipeptidyl peptidase-4 inhibitors, glucagon-likepeptide-1 agonists, biguanides, sodium-glucosecotransporter-2 inhibitors, thiazolidinediones, and alpha-glucosidase inhibitors. ACEi/ARBs: angiotensin-converting enzyme inhibitors/angiotensin II receptor blockers.

**Table 2:** Characteristics of 11 eyes examined with optical coherence tomography angiography.

Variable	All eyes (*n* = 11)	Eyes without diabetic retinopathy^*∗*^ (*n* = 4)	Eyes with diabetic retinopathy (*n* = 7)	*p* value
30° nonperfusion, mean (SD)	6.58% (4.23%)	4.41% (1.30%)	7.81% (4.91%)	0.216
60° nonperfusion, mean (SD)	16.90% (7.71%)	11.15% (2.49%)	20.18% (7.83%)	0.057
30° vessel density, mean (SD)	45.12% (1.81%)	45.55% (2.52%)	44.87% (1.46%)	0.756
60° vessel density, mean (SD)	41.54% (3.69%)	43.28% (2.48%)	40.54% (3.56%)	0.154

^*∗*^Asterisk indicates eyes from participants with diabetes but without diabetic retinopathy as defined by Early Treatment Diabetic Retinopathy Study criteria.

**Table 3:** Correlations of retinal nonperfusion and retinal vessel density with estimated glomerular filtration rate (eGFR) and urine albumin-creatinine ratio.

eGFR							Urine albumin-to-creatinine ratio					
	All eyes (*n* = 11)		Eyes without diabetic retinopathy (*n* = 4)		Eyes with diabetic retinopathy (*n* = 7)		All eyes (*n* = 11)		Eyes without diabetic retinopathy (*n* = 4)		Eyes with diabetic retinopathy (*n* = 7)	
	*R*(correlation)	*p*value	*R*(correlation)	*p*value	*R*(correlation)	*p* value	*R*(correlation)	*p*value	*R*(correlation)	*p*value	*R*(correlation)	*p*value
30° nonperfusion	−0.686	0.004	−0.032	0.979	−0.885	0.008	−0.167	0.481	0.288	0.712	0.113	0.809
60° nonperfusion	−0.457	0.057	0.217	0.783	−0.844	0.017	−0.315	0.183	−0.443	0.557	−0.051	0.913
30° vessel density	0.267	0.267	−0.117	0.883	0.809	0.027	0.093	0.695	0.109	0.891	0.289	0.530
60° vessel density	0.305	0.205	−0.205	0.795	0.653	0.112	0.278	0.240	0.396	0.604	0.273	0.554

## Data Availability

The data used to support this study are available from the corresponding author upon request.
